# Association of Alzheimer's Disease Blood Biomarkers With Sarcopenia Incidence and Progression: A 12‐Year Population‐Based Study

**DOI:** 10.1002/jcsm.13835

**Published:** 2025-06-09

**Authors:** Chiara Ceolin, Caterina Gregorio, Alice Margherita Ornago, Giulia Grande, Martina Valletta, Caterina Trevisan, Adrián Carballo Casla, Giuseppe Sergi, Amaia Calderón‐Larrañaga, Davide Liborio Vetrano

**Affiliations:** ^1^ Department of Neurobiology, Care Sciences and Society Karolinska Institutet and Stockholm University, Aging Research Center Stockholm Sweden; ^2^ Geriatrics Division, Department of Medicine (DIMED) University of Padua Padua Italy; ^3^ School of Medicine and Surgery University of Milano‐Bicocca Milan Italy; ^4^ Stockholm Gerontology Research Center Stockholm Sweden; ^5^ Department of Medical Sciences University of Ferrara Ferrara Italy; ^6^ Center for Networked Biomedical Research in Epidemiology and Public Health (CIBERESP) Madrid Spain

**Keywords:** Alzheimer disease, blood biomarkers, neurodegeneration, older adults, protein intake, sarcopenia

## Abstract

**Background:**

Sarcopenia is a complex and multifactorial condition, and recent studies have explored the role of neurological markers in its diagnosis and prediction. Although associations have been identified between reduced muscle strength, slow walking speed and elevated neurofilament levels (NfL), long‐term evidence and sex‐based differences in muscle health and sarcopenia remain underexplored. This study investigates the relationship between baseline blood biomarkers of Alzheimer's disease (AD) and long‐term sarcopenia trajectories in a Swedish cohort of older adults, while also examining potential sex‐based differences.

**Methods:**

The study analysed 2291 participants aged ≥ 60 years (61.5% females) over a 12‐year follow‐up, classifying sarcopenia into three stages (no, probable and confirmed sarcopenia) using modified EWGSOP2 criteria. Muscle strength was assessed via handgrip or chair stand tests and muscle mass via calf circumference. Baseline data on AD biomarkers were collected. Latent class mixed models identified two sarcopenia trajectories: one with early progression accelerating around age 70 years and another with later progression accelerating after age 80 years, observed in both sexes. Regression analyses examined the associations between AD biomarkers, sarcopenia progression speed and incidence.

**Results:**

Probable and confirmed sarcopenia were more prevalent in females (28.2% vs. 14.1% and 7.6% vs. 6.1%, respectively; *p* < 0.001). All AD biomarkers showed significantly different distributions across the three sarcopenia stages. Analysis revealed that only p‐tau181 (OR 1.24 [1.09; 1.42], *p* = 0.002) and NfL (OR 1.56 [1.30; 1.91], *p* < 0.001) were independently associated with worse sarcopenia trajectories. These associations remained significant in individuals over 78 years (p‐tau181: OR 1.32 [1.11; 1.59], *p* = 0.003; NfL: OR 1.77 [1.40; 2.28], *p* < 0.001) and in males (p‐tau181: OR 1.39 [1.14; 1.73], *p* = 0.003; NfL: OR 1.38 [1.11; 1.82], *p* < 0.001). In females, only NfL remained significantly associated. NfL was significantly linked to sarcopenia development (HR 1.20 [1.10; 1.30], *p* < 0.001), with similar findings for females (HR 1.40 [1.20; 1.63], *p* < 0.001) and older individuals (HR 1.35 [1.15; 1.58], *p* < 0.001). Notably, both NfL and p‐tau181 were significantly associated with sarcopenia incidence in younger participants (< 78 years) and in males, independent of dietary patterns.

**Conclusions:**

Our study, unique for its long follow‐up duration, explores the relationship between sarcopenia and neurodegeneration biomarkers, highlighting the role of p‐tau181 and NfL in the progression of the condition. These biomarkers could potentially serve as indicators for the early detection of sarcopenia, particularly in older adults and males, offering insights that may contribute to personalized screening and targeted interventions.

## 1 Introduction

Sarcopenia, characterized by the gradual decline in muscle mass and quality, poses a major obstacle to healthy ageing, affecting around 40% of individuals over 70 worldwide, with this figure expected to rise significantly by 2050 [1]. The rate of skeletal muscle loss varies greatly among individuals of the same age [2]. Sarcopenia is a leading cause of mobility limitations and disability in older adults, increasing their risk of injurious falls, hospitalizations and mortality [3, 4]. Therefore, early identification and prediction of sarcopenia is crucial for implementing timely interventions to maintain independence and quality of life in older adults. According to the latest guidelines of the ‘European Working Group on Sarcopenia in Older People 2’ (EWGSOP2), sarcopenia progresses through three stages: it begins with an initial reduction in muscle strength (probable sarcopenia), then develops with a concomitant loss of muscle mass and strength (confirmed sarcopenia), and it finally impacts functional abilities (severe sarcopenia) [5].

Sarcopenia has gained substantial attention in recent years, which has led to incipient efforts to better understand its complex and multifactorial nature. Among the risk factors contributing to accelerated muscle ageing, lifestyle‐related factors such as physical inactivity and poor nutrition, along with physiological factors like metabolic and chronic diseases or hormonal changes, have been identified as major players [6, 7]. In addition, several mechanisms have been recognized to contribute to the pathophysiology of sarcopenia, including low‐grade inflammation associated with chronic conditions and accelerated ageing phenotypes, mitochondrial dysfunction and oxidative stress [8, 9]. Only recently, along with novel insights into the role of the neurological system in the regulation of skeletal muscle health in adulthood, the exploration of the contribution of neurological markers as potential sources of sarcopenia diagnosis and prognosis has begun [10]. Initially, myofibre atrophy was thought to be the primary hallmark of the decline in muscle strength associated with ageing [10]. However, increasing evidence suggests that this process might be influenced to a greater extent by neurodegenerative changes [11, 12, 13, 14]. Neurodegeneration can disrupt neuromuscular signalling, impairing muscle fibre recruitment and fostering muscle atrophy [15]. Among neurodegenerative conditions, Alzheimer disease (AD) pathology stands out as one of the most thoroughly studied and represents a key therapeutic target for emerging pharmacological interventions. Although these treatments currently have limitations, they show promise in slowing the progression of cognitive symptoms. Investigating the potential link between AD pathology and sarcopenia could open new avenues for therapeutic applications, offering opportunities to mitigate sarcopenia's impact by leveraging advances in AD research. Recent studies has reported associations between reduced grip strength, slow walking speed, severe sarcopenia and increased levels of blood neurofilaments (NfL), as well as positive correlations between better muscle strength values and lower plasma levels of amyloid beta 40 (Aβ40) and total tau (t‐tau) [11, 12, 13, 14]. These biomarkers, measurable in cerebrospinal fluid and blood, provide valuable insights into neuronal health and the progression of neurodegeneration, offering a potential bridge between muscle health and neurodegenerative processes. Despite these promising findings, the evidence remains limited by the lack of long‐term studies, which hinders causal inference and restricts our understanding of the neurological mechanisms underlying sarcopenia. Moreover, sex‐based differences have not been adequately investigated, leaving an important gap in the knowledge of how sarcopenia and muscle health are influenced by biological sex. Addressing these gaps is crucial for developing a more comprehensive understanding of the interplay between neurodegeneration and sarcopenia. In this context, our aim was to explore the association between baseline blood‐based biomarkers of AD and long‐term sarcopenia trajectories within a Swedish population‐based cohort of older adults. Additionally, we sought to investigate sex‐based differences in these associations, considering the potential impact of biological sex on muscle health and sarcopenia progression. We hypothesized that older individuals with an increased burden of AD pathology, as indicated by altered concentrations of specific blood biomarkers, also experience a more rapid progression of sarcopenia.

## 2 Materials and Methods

### 2.1 Study Population

This longitudinal observational study utilizes data from the Swedish National Study on Aging and Care in Kungsholmen (SNAC‐K), encompassing a Swedish population aged ≥ 60 years. Participants were selected through random stratified sampling, within the neighbourhood of Kungsholmen, including the following age cohorts: 60, 66, 72, 78, 81, 84, 87, 90, 93, 96 and 99+ years. The initial assessment, conducted from 2001 to 2004, achieved a participation rate of 73.3%. The youngest cohorts (ages 60–72 years) have been followed every 6 years, while the older cohorts (age ≥ 78 years) have been followed every 3 years. This study incorporates data from the first five waves of SNAC‐K, spanning a maximum of 12 years. The SNAC‐K study received approval from the Swedish Ethical Review Authority, and written informed consent was obtained from participants or their next of kin. From an initial sample of 3363 individuals, those with dementia, Parkinson's disease, multiple sclerosis, those living in institutions (*N* = 341) and participants with incomplete data (*N* = 731) on muscle mass, muscle strength or biomarker measurements were excluded, resulting in a final sample of 2257 participants (Figure S1). In detail, the exclusion of patients with dementia followed DSM‐IV criteria through a three‐step process. Initially, a physician made a preliminary diagnosis based on health history, physical and neurological examinations and cognitive tests such as the MMSE and other assessments. A second independent physician reviewed the data for a separate diagnosis, and discrepancies were resolved by an external neurologist. For deceased participants without a prior dementia diagnosis, medical records were reviewed retrospectively. The Swedish Cause of Death Register was also cross‐referenced with SNAC‐K to identify dementia cases using ICD‐10 codes.

### 2.2 Study Variables

#### 2.2.1 Sarcopenia

The presence of sarcopenia at baseline and at each follow‐up wave was assessed using a modified version of the revised criteria from the EWGSOP2 [5]. This assessment included evaluations of muscle strength, muscle mass and physical performance. The methodology for assessing sarcopenia in our participants has been previously detailed [16]. In brief, muscle strength was estimated using the highest score obtained from the handgrip test performed with each hand. In cases where handgrip data were unavailable, the sit‐to‐stand test results was utilized. Low muscle strength was defined as a handgrip strength of less than 27 kg for male and 16 kg for females, or a time exceeding 15 s for five sit‐to‐stand repetitions. Low muscle mass was defined as a calf circumference below the 20th percentile of our sample [17, 18], corresponding to < 34 cm in males and < 32 cm in females. These thresholds align with the cut‐off values for moderately to severely low calf circumference proposed in a recent study [19]. Low physical performance was defined as a walking speed of 0.8 m/s or less, measured over a distance of 6 m, or, for participants who self‐reported walking slowly or were evaluated at home, over a distance of 2.4 m. Following the EWGSOP2 algorithm, participants were categorized into three groups: ‘no sarcopenia’ for people with normal muscle strength and mass; ‘probable sarcopenia’ for those with low muscle strength but normal muscle mass; and ‘sarcopenia’—encompassing both confirmed and severe sarcopenia—for participants with low muscle strength and mass, regardless of physical performance. The decision to consider severe sarcopenia alongside confirmed sarcopenia was driven by the limited number of participants presenting with severe sarcopenia.

#### 2.2.2 Blood biomarkers

Peripheral venous blood samples were collected at baseline. The methodology for assessing blood biomarkers in the study participants has been previously outlined [20]. Briefly, following centrifugation, serum aliquots were preserved at Karolinska Institutet BioBank at −80°C in cryogenic storage vials until analysis. Protein quantification for AD blood biomarkers was conducted at the Affinity Proteomics Stockholm Unit (SciLifeLab). Serum NfL light chain and glial fibrillary acidic protein (GFAP) levels were determined using the Simoa Neuro 2‐plex B Kit. Serum Aβ40, Aβ42 and t‐tau were evaluated using the Simoa Neuro 3‐plex A Kit. Serum phosphorylated‐tau181 (p‐tau181) was assessed with the Simoa pTau‐181 Advantage V2 Kit. For each kit, 25 μL of sample was diluted 1:4, and the assays were conducted following the manufacturer's instructions. The Quanterix instrument provided average enzyme per bead (AEB) values for calibrators, controls and samples. Curve fitting, concentration extrapolation and graphical representation were automatically managed within the Quanterix SR‐X software using the calibrators and a four‐parameter logistic (4PL) curve fit. Data below the limit of detection were handled by imputing a value of 0 using a single‐value imputation strategy for the following measurements: 6 for Aβ42, 15 for p‐tau181 and 15 for t‐tau.

#### 2.2.3 Covariates

The following sociodemographic data were collected for each participant: age, sex, education level (classified as elementary, high school diploma or university) and cohabitant status (classified as having a partner or living alone). Regarding lifestyle characteristics, we considered smoking habits, alcohol consumption and physical activity levels. We categorized smoking habits as never smoker, former smoker or current smoker and classified alcohol consumption as never or occasional drinker, light to moderate (1–14 alcohol unit per week for males and 1–7 alcohol unit per week for females) or heavy (15 or more drinks per week for males and 8 or more drinks per week for females). Physical activity levels were classified into three groups based on frequency and intensity, following the recommendations of the World Health Organization and American College of Sports Medicine [21, 22]: (a) inadequate, 2–3 times or less per month of light and/or moderate/intense exercise; (b) health‐enhancing, light exercise several times per week; and (c) fitness‐enhancing, moderate/intense exercise several times per week. We also gathered information on three ancillary variables. Body mass index (BMI) at baseline was calculated as measured weight divided by measured height squared (kg/m^2^). Global cognitive performance was assessed using the Swedish version of the Mini‐Mental State Examination (MMSE). Additionally, information on 60 chronic disease categories, and their total number, was considered [23] (please refer to Supporting information S1). We collected dietary data with a validated 98‐item Food Frequency Questionnaire (FFQ) [24], where participants reported their annual frequency of food and drink intake on a nine‐point scale. Portion sizes were estimated using photographs, and nutrient intake was calculated via Swedish National Food Agency tables. Mediterranean diet adherence was assessed using the Trichopoulou method [25], grouping FFQ items into nine categories and calculating daily consumption averages. Categories were scored 0 or 1 based on the sex‐specific median, with higher scores for beneficial components (e.g., vegetables and fish) and lower consumption of detrimental ones (e.g., meat and dairy). Alcohol intake was scored if within specified limits for men and women. The Mediterranean Diet Score (MDS) ranged from 0 to 9.

### 2.3 Statistical Analysis

Baseline characteristics of the sample were reported as means (standard deviations [SD]), medians (interquartile ranges [IQR]), or counts (%). Characteristics between males and females were compared through Student's *t*‐tests and χ^2^ or Fisher's exact tests. We used the Kruskal–Wallis test to compare biomarker concentrations across the three stages of sarcopenia. Blood biomarkers were transformed into *z*‐scores based on the baseline mean and SD of the entire sample, facilitating the comparison between coefficients.

Latent sarcopenia trajectories over the 12‐year follow‐up were delineated using latent class mixed models [26]. These models identify distinct latent classes of subjects based on similarities in patterns of change in muscle strength and mass over time. Specifically, threshold mixed models were employed to describe the relationship between each level of the ordinal sarcopenia variable and the underlying latent process. Age was used as the time scale to obtain sarcopenia trajectories over the older age period. Two latent trajectories were selected as the optimal number by evaluating the Bayesian information criterion (BIC). A mixed model with random intercept and random slope for age was used. In both fixed and random effects, a natural cubic spline with two degrees of freedom was used for age to account for the nonlinear sarcopenia trajectories in older life. Additionally, an interaction term between age and sex was included as a fixed effect in both the mixed model and the latent process mixed model, to account for sex differences in sarcopenia trajectories and latent sarcopenia trajectories. Participants were assigned to the latent class for which they had the highest posterior probability of membership (i.e., the most likely class). To examine the association between AD and neurodegeneration biomarkers and speed of sarcopenia progression, we conducted logistic regression analyses. The outcome variable was the membership to the earlier sarcopenia progression trajectory. For each AD and neurodegeneration biomarkers, odds ratios (ORs) and 95% confidence intervals (CIs) were calculated to assess the likelihood of earlier sarcopenia progression per 1 SD increase in biomarker levels. Additionally, Cox proportional hazards regression models were used to assess the independent association of AD and neurodegeneration biomarkers with sarcopenia incidence. Logistic and Cox regression models were adjusted for baseline age, sex, education, smoking and alcohol consumption, chronic diseases (e.g., cardiovascular disease, cerebrovascular disease, diabetes, chronic kidney disease) and physical activity. Moreover, the logistic and Cox regression models were stratified by sex and age (≥ 78 and < 78 years) and applied to a subgroup of participants with a complete dietary pattern assessment. To minimize potential confounding factors and better isolate the independent association between biomarkers and sarcopenia incidence, sensitivity analyses were conducted using Cox regression models. These analyses followed two approaches: excluding participants with probable sarcopenia at baseline and further excluding those with musculoskeletal disorders at baseline. All sensitivity analyses were based on Model 1 (adjusted for sex, age and education level) and Model 2 (further adjusted for smoking, alcohol consumption, diabetes, cardiovascular diseases, cerebrovascular diseases, chronic kidney disease and physical activity). For individuals with complete dietary pattern assessments, Model 3 was also used (additionally adjusted for energy and protein intake, as well as adherence to the Mediterranean diet). A two‐tailed *p* value < 0.05 was considered statistically significant in all analyses. The statistical analyses were performed using R version 4.3.2 (2023‐10‐31), packages: ‘lcmm’, ‘survival’ and ‘ggplot2’.

## 3 Results

The study population had a median age of 72.1 years (IQR range: 60.8–78.8), with the majority being female (61.5%). Table 1 presents the baseline characteristics of the participants for the entire sample and by sex. Male, on average, were younger than females and had higher prevalence of overweight, greater educational attainment and higher proportions of smoking and alcohol consumption. Regarding chronic diseases, females had higher prevalence of chronic kidney disease. Conversely, males had higher proportions of heart diseases. In terms of biomarker levels, males had lower values for all biomarkers except p‐tau181, which was higher compared with females.

**TABLE 1:** Baseline descriptive characteristics of the study population.

Variables	Overall (*n* = 2291)	Male (*n* = 882) 38.5%	Female (*n* = 1409) 61.5%	*p*
Age (years)	72.1 [60.8; 78.8]	66.5 [60.6; 78.2]	72.3 [61.0; 81.2]	< 0.001
Level of education				< 0.001
Elementary	339 (14.8%)	117 (13.3%)	222 (15.8%)	
High school	1124 (49.1%)	368 (41.7%)	756 (53.7%)	
University or more	828 (36.1%)	397 (45.0%)	431 (30.6%)	
Civil status				< 0.001
Unpartnered	1158 (50.6%)	276 (31.3%)	882 (62.7%)	
Partnered	1131 (49.4%)	606 (68.7%)	525 (37.3%)	
Smoking habit				< 0.001
Never	1017 (44.6%)	298 (32.8%)	728 (52.0%)	
Former or current	1264 (55.4%)	591 (67.2%)	673 (48%)	
Alcohol consumption				< 0.001
No or occasional	710 (31.1%)	191 (21.8%)	519 (36.9%)	
Light to moderate	1717 (51.3%)	586 (66.8%)	585 (41.6%)	
Heavy	401 (17.6%)	100 (11.4%)	301 (21.4%)	
Physical activity level				0.22
Low	620 (27.1%)	226 (25.6%)	394 (28.0%)	
Intermediate or high	1671 (72.9%)	656 (74.4%)	1015 (72.0%)	
BMI (kg/m^2^)				< 0.001
< 18.5	35 (1.6%)	7 (0.8%)	28 (2.1%)	
18.5–24.9	948 (43.5%)	309 (36.1%)	639 (48.3%)	
25–29.9	898 (41.2%)	416 (48.6%)	482 (36.4%)	
≥ 30	299 (13.7%)	124 (14.5%)	175 (13.2%)	
MMSE				0.001
MMSE ≥ 27	2067 (90.4%)	820 (93.1%)	1247 (88.7%)	
MMSE < 27	220 (9.6%)	61 (6.9%)	159 (11.3%)	
Chronic diseases				
Number of chronic diseases	3.72 (2.32)	3.48 (2.30)	3.86 (2.32)	0.98
Atrial fibrillation	189 (8.2%)	86 (9.8%)	103 (7.3%)	0.04
Heart failure	168 (7.3%)	66 (7.5%)	102 (7.2%)	0.83
Ischaemic heart diseases	305 (13.3%)	138 (15.6%)	167 (11.9%)	0.01
Cerebrovascular diseases	125 (5.5%)	45 (5.1%)	80 (5.7%)	0.57
Chronic kidney diseases	730 (31.9%)	194 (22.0%)	536 (38.0%)	< 0.001
Diabetes	200 (8.7%)	109 (12.4%)	91 (6.5%)	< 0.001
Inflammatory arthropathies	86 (3.8%)	33 (3.7%)	53 (3.8%)	0.90
Osteoarthrosis	291 (12.7%)	90 (10.2%)	201 (14.3%)	0.005
Other MSK and joint diseases	128 (5.6%)	50 (5.7%)	78 (5.5%)	0.93
Number of drugs	3.00 [1.00; 5.00]	2.00 [0.00; 4.00]	3.00 [2.00; 6.00]	< 0.001
AD blood biomarkers				
Aβ42/40	0.06 [0.05; 0.07]	0.06 [0.05; 0.06]	0.06 [0.05; 0.07]	0.03
t‐Tau (pg/mL)	0.83 [0.55; 1.18]	0.80 [0.52; 1.14]	0.86 [0.55; 1.21]	0.03
p‐Tau181 (pg/mL)	1.17 [0.75; 1.78]	1.23 [0–75; 1.87]	1.12 [0.74; 1.75]	0.05
NfL (pg/mL)	17.85 [12.50; 27.81]	16.95 [12.21; 25.57]	18.53 [12.76; 29.18]	0.001
GFAP (pg/mL)	120.69 [79.94; 187.67]	102.61 [68.48; 152.44]	133.08 [91.40; 211.04]	< 0.001

*Note:* Numbers are expressed as mean (standard deviation), median [interquartile range] or number (percentage %) as appropriate. *p* values refer to the comparison between males and females. Missing data: civil status (*n* = 2), alcohol consumption (*n* = 9), smoking habit (*n* = 9) and MMSE (*n* = 4).

Abbreviations: AD, Alzheimer's disease; Aβ40, 40‐aminoacid β amyloid peptide; Aβ42, 42‐aminoacid β amyloid peptide; BMI, body mass index; GFAP, glial fibrillary acidic protein; MMSE, mini‐mental state examination; MSK, musculoskeletal; NfL, neurofilament light; p‐Tau181, phosphorylated tau 181; t‐Tau, total tau.

**FIGURE 1:**
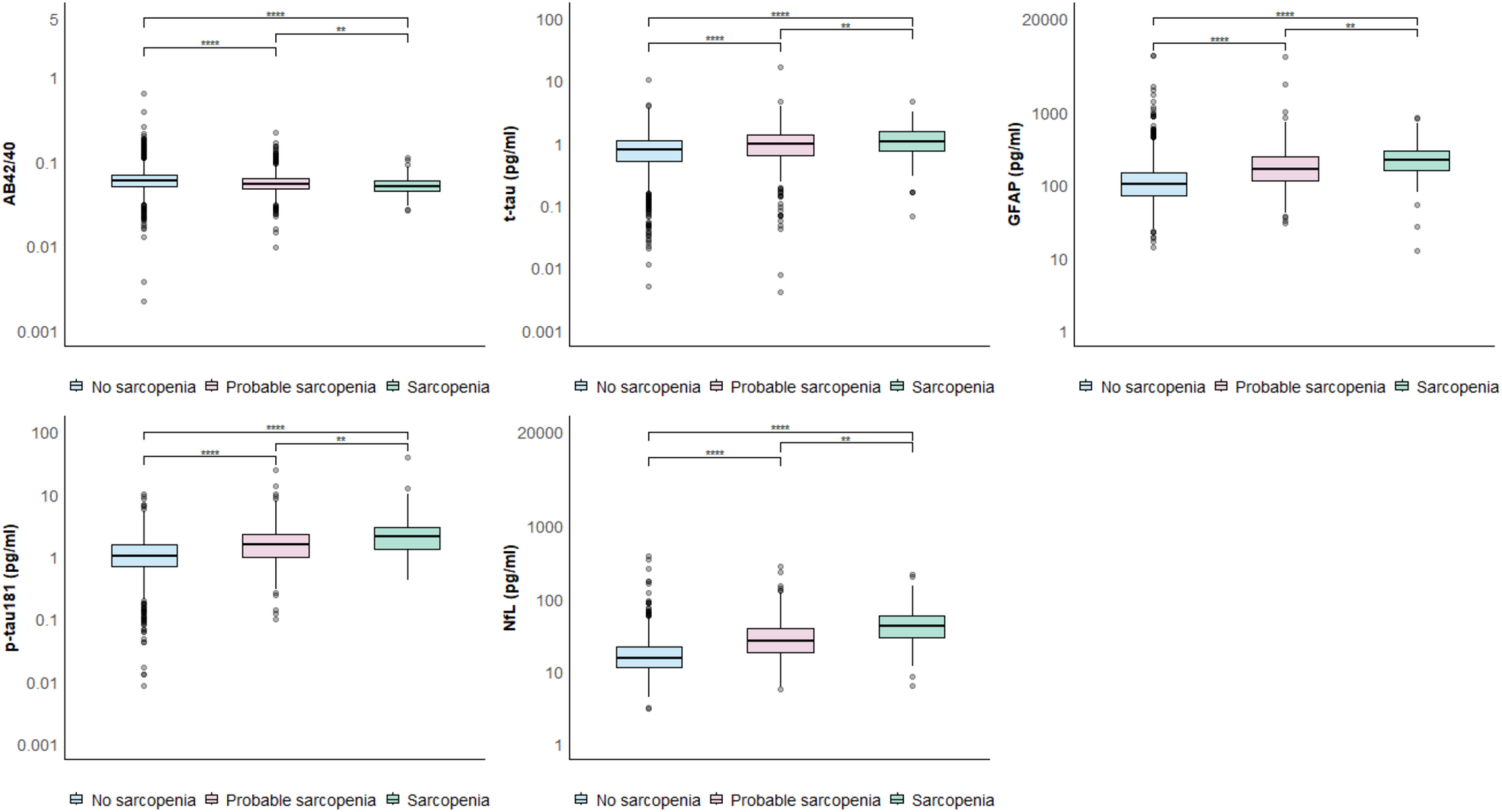
Distribution of the blood biomarkers of Alzheimer's disease by sarcopenia categories at baseline. *Notes:* Box plots show the median (central line) and interquartile range (box) as well as the 2.5th and 97.5th percentiles (whiskers). *p* values are derived from Kruskal–Wallis test; *: < 0.05; **: < 0.01; ***: < 0.001; ****: < 0.0001. Aβ42/40 amyloid‐beta 42/40; GFAP, glial fibrillary acidic protein; NfL, neurofilament light chain; p‐tau181, phosphorylated‐tau181; t‐tau, total‐tau.

Regarding sarcopenia assessment, females had a significantly higher prevalence of low muscle strength compared with males (73.9% vs. 26.1%, *p* < 0.001) (Table 2). This difference contributed to substantial sex disparities in the prevalence of both probable and confirmed sarcopenia (28.2% vs. 14.1% and 7.6% vs. 6.1%, respectively, *p* < 0.001). However, no significant difference was observed between males and females in the prevalence of low muscle mass (59.1% vs. 40.9%, *p* = 0.29). All biomarkers showed significantly different distributions across the three stages of sarcopenia: Participants with probable or confirmed sarcopenia had elevated concentrations of all biomarkers except for the Aβ42/40 ratio, which was lower in these groups (Figure 1).

**TABLE 2:** Muscle strength, mass and physical performance of the study population at baseline.

Variables	Overall (*n* = 2291)	Male (*n* = 882)	Female (*n* = 1409)	*p*
Grip strength (kg_f_)	24.58 [18.05; 34.85]	37.72 [30.28; 43.44]	19.68 [15.50; 24.07]	< 0.001
Chair stand test (s)	13.00 [10.00; 20.00]	12.00 [9.00; 17.00]	14.00 [11.00; 24.00]	< 0.001
Calf circumference (cm)	36.23 (3.57)	37.12 (3.35)	35.67 (3.59)	< 0.001
Walking speed (m/s)	0.91 (0.53)	1.04 (0.57)	0.83 (0.50)	< 0.001
Presence of sarcopenia				< 0.001
No sarcopenia	1608 (70.2%)	704 (79.8%)	904 (64.2%)	
Probable	522 (22.8%)	124 (14.1%)	398 (28.2%)	
Confirmed sarcopenia	161 (7.0%)	54 (6.1%)	107 (7.6%)	
Sarcopenia latent profile				< 0.001
Early progression	1194 (52.1%)	421 (47.7%)	773 (54.9%)	
Late progression	1097 (47.9%)	461 (52.3%)	636 (45.1%)	

*Note:* Numbers are expressed as mean (standard deviation), median [interquartile range] or number (percentage) as appropriate. *p* values refer to the comparison between males and females.

**FIGURE 2:**
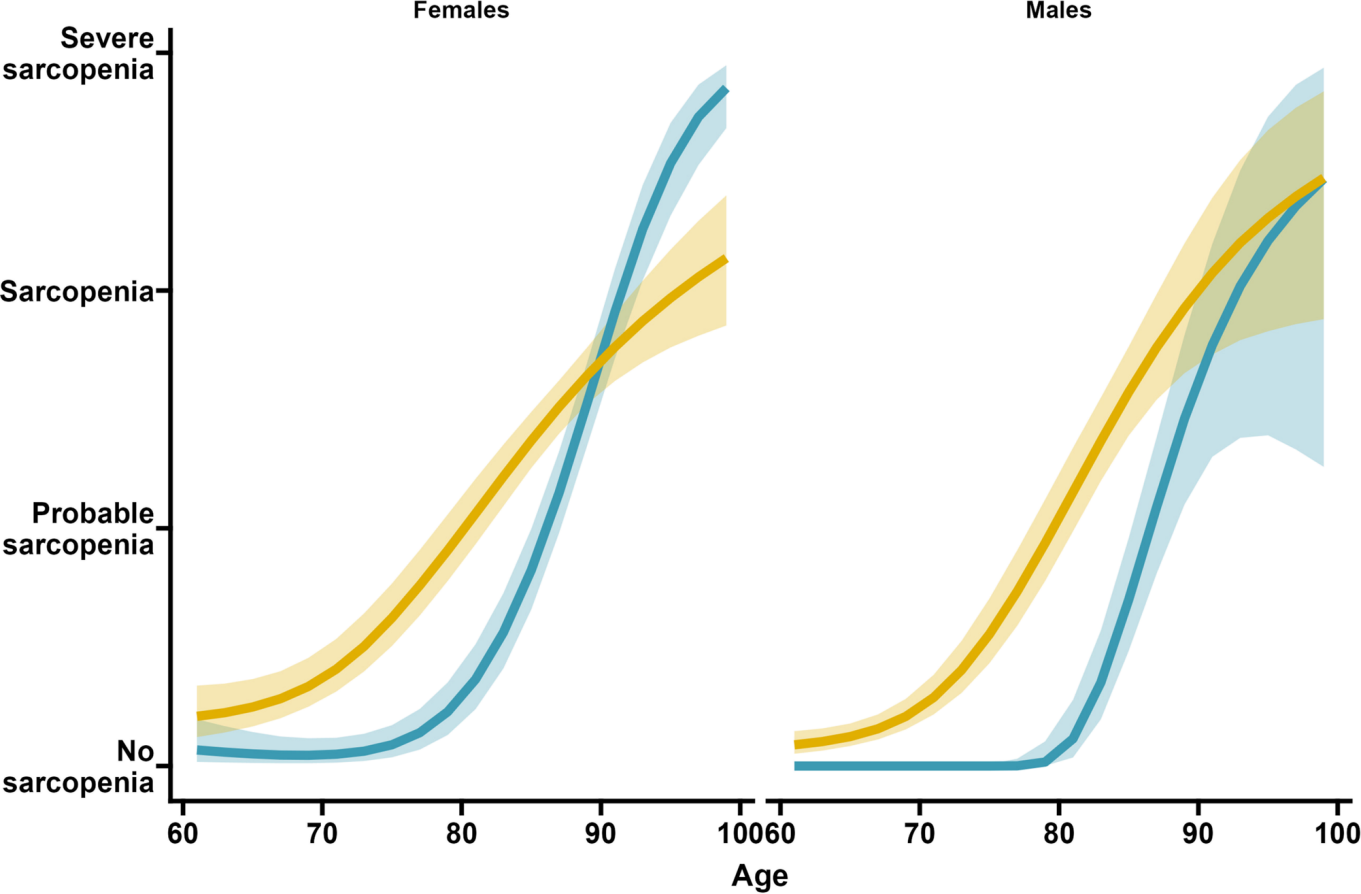
Latent profiles of sarcopenia progression stratified by sex: colour‐coded representation of early progression trajectory (yellow) and late progression trajectory (blue). *Notes*: The figure displays the median (solid line) and 95% confidence interval (shade).

We identified two distinct latent profiles of sarcopenia progression over time: the first, characterized by an earlier progression with an acceleration at age 70 years, and the second, showing a later progression and an acceleration after 80 years old, in both males and females (Figure 2).

The analysis of the association between AD biomarkers and earlier sarcopenia progression revealed that only p‐tau181 (OR 1.24 [1.09; 1.42], *p* = 0.002) and NfL (OR 1.56 [1.30; 1.91], *p* < 0.001) were independently associated with the worse sarcopenia trajectory, also after adjustment for baseline age, sex, education, smoking and alcohol consumption, chronic diseases and physical activity (Table 3). Upon stratification, these associations remained significant in individuals over 78 years old (Table S1) and in males (Table S2), whereas in females, only NfL remained a significantly associated. The same results were also confirmed when examining the incidence of probable sarcopenia (data not shown). The association between p‐tau181 and NfL remained significant even after adjusting for dietary variables, particularly in older individuals, with sex‐specific differences observed for p‐tau181 and Nfl (Tables S3–S5). We then examined the association between biomarkers and sarcopenia incidence, in a subgroup of participants who were sarcopenia‐free at baseline. As shown in Table 4, only NfL was significantly associated with the development of sarcopenia (HR 1.20 [1.10; 1.30], *p* < 0.001). When stratifying by age and sex, this was also the case in women and older individuals. Conversely, both NfL and p‐tau181 were significantly associated with sarcopenia incidence in younger participants (under 78 years old) and in males (please see Tables S6 and S7, respectively).

**TABLE 3:** Associations between Alzheimer's disease biomarkers (per 1 SD increase) and early sarcopenia progression (*n* = 2291).

	Model 1		Model 2	
	OR (95% CI)	*p*	OR (95% CI)	*p*
Aβ42/40	1.02 (0.94; 1.11)	0.70	1.02 (0.94; 1.12)	0.60
t‐Tau	1.07 (0.98; 1.17)	0.20	1.03 (0.95; 1.14)	0.50
p‐Ttau181	1.30 (1.14; 1.49)	< 0.001	1.24 (1.09; 1.42)	0.002
NfL	1.67 (1.40; 2.02)	< 0.001	1.56 (1.30; 1.91)	< 0.001
GFAP	1.05 (0.96; 1.17)	0.30	1.05 (0.96; 1.16)	0.30

*Note:* Model 1 is adjusted for sex, age and education; Model 2 is additionally adjusted for smoking and alcohol consumption, physical activity, as well as comorbidities including diabetes, heart, cerebrovascular and chronic kidney disease.

Abbreviations: Aβ40, 40‐aminoacid β amyloid peptide; Aβ42, 42‐aminoacid β amyloid peptide; GFAP, glial fibrillary acidic protein; NfL, neurofilament light; t‐Tau, total tau; p‐Tau181, phosphorylated tau 181.

**TABLE 4:** Association between Alzheimer's disease biomarker and sarcopenia incidence (*n* = 2291).

	Model 1		Model 2	
	HR (95% CI)	*p*	HR (95% CI)	*p*
Aβ42/40	1.07 (0.98; 1.16)	0.12	1.07 (0.99; 1.16)	0.10
t‐Tau	0.93 (0.81; 1.06)	0.30	0.91 (0.79; 1.04)	0.20
p‐Tau181	1.16 (1.00; 1.34)	0.047	1.15 (0.99; 1.34)	0.067
NfL	1.19 (1.10; 1.29)	< 0.001	1.20 (1.10; 1.30)	< 0.001
GFAP	0.99 (0.86; 1.14)	0.90	0.98 (0.85; 1.14)	0.80

*Note:* Model 1 is adjusted for sex, age and education; Model 2 is additionally adjusted for smoking and alcohol consumption, physical activity, as well as comorbidities including diabetes, heart, cerebrovascular and chronic kidney disease.

Abbreviations: Aβ40, 40‐aminoacid β amyloid peptide; Aβ42, 42‐aminoacid β amyloid peptide; GFAP, glial fibrillary acidic protein; NfL, neurofilament light; t‐Tau, total tau; p‐Tau181, phosphorylated tau 181.

In sensitivity analyses, after excluding participants with probable sarcopenia, no biomarkers were associated with sarcopenia incidence. When stratifying for age and sex, p‐tau181 and GFAP emerged as the markers significantly associated with sarcopenia incidence in individuals over 78 years old and in males (Tables S8–S10). After excluding also participants with musculoskeletal disorders, NfL continued to be significantly associated with incident sarcopenia in the whole sample (Table S11), younger and older participants, and in females (Tables S12 and S13). p‐Tau181 showed a significant association in younger participants and in men. Finally, when adjusting for energy and protein intake and adherence to the Mediterranean diet, p‐tau181 and NfL emerged as the only biomarkers significantly associated with incident sarcopenia, particularly in older individuals. Among these, only NfL remained significant in both males and females (Tables S14–S16).

## 4 Discussion

This study explored the complex interactions between skeletal muscle health and the nervous system by investigating the relationship between AD blood‐based biomarkers and sarcopenia in individuals aged 60 year or older. Among the studied biomarkers, NfL and p‐tau181 were associated with the risk of earlier sarcopenia development and progression, a relationship that was particularly evident in the oldest participants and males. However, among participants free from sarcopenia at baseline, only NfL was significantly associated with incident sarcopenia, while p‐tau181 showed an association only in younger male individuals. These results were independent of dietary patterns, including protein and energy intake. Sarcopenia is a multifaceted and heterogeneous geriatric syndrome. As such, focusing on one or a few individual biological mechanisms, and consequently disease biomarkers, may fall short in capturing the underlying biological complexity of this condition [27]. In recent years, several potential muscular and extra‐muscular biomarkers of sarcopenia have been identified, including inflammatory, nutritional and endocrine markers [9]. Although the causes of sarcopenia are thought to be multifactorial, growing evidence indicates that neurological processes play a significant role in regulating skeletal muscle health [13, 28]. This could be primarily related to two mechanistic pathways. First, sarcopenia and neurodegeneration may share common underlying causes, as for example chronic diseases and inflammaging [8]. As people age, increased pro‐inflammatory cytokine production from dysregulated metabolic processes and deleterious lifestyle changes contribute to functional decline in organs like the brain, triggering inflammation and neurodegeneration [29]. Second, motor neuron degeneration, as observed in various neurodegenerative diseases, disrupts the communication between the nervous system and muscles [30]. Without adequate stimulation, muscle fibres undergo atrophy, characterized by a reduction in size and overall muscle volume [30], resulting in a decrease in muscle mass and strength. Furthermore, research has shown that muscle fibres can display both atrophic and hypertrophic changes, depending on the severity of motor neuron loss. For instance, severe cases of spinal muscular atrophy often reveal muscle biopsies with rounded, atrophic fibres interspersed with hypertrophic fibres [31]. This pattern indicates cycles of denervation and failed attempts at reinnervation, as continuous neuronal loss hampers successful muscle fibre regeneration. Furthermore, neurodegeneration can induce structural changes at the neuromuscular junction, where motor neurons connect with muscle fibres [32]. These defects at the neuromuscular junction level can precede the complete loss of motor neurons and further impair muscle function, exacerbating muscle atrophy [32]. Given these factors, it is reasonable to hypothesize that a bidirectional relationship exists between neurodegeneration and sarcopenia.

AD biomarkers may offer new insights into the mechanisms underlying sarcopenia. Recent studies have explored the association between NfL and sarcopenia, demonstrating that elevated levels of this biomarker are linked to poorer muscle and functional performance, as well as more severe stages of sarcopenia [10, 12, 13]. Research by Capo et al. showed a significant negative correlation between NfL levels and tests such as grip strength, gait speed and the chair‐stand test, associations being especially pronounced in males [10]. However, these studies are based on small samples sizes [10] or heterogeneous populations that include both young adults and older individuals and therefore do not provide a specific understanding of the sarcopenia dynamics related to ageing [10, 13]. Furthermore, most of these studies have only analysed individual aspects of sarcopenia, such as muscle strength, body composition or performance tests, rather than the condition as a whole [10, 13]. In contrast, our study differs in several important ways: (1) It specifically focuses on an older adult population, ensuring that the results are more applicable to the age group most affected by sarcopenia; (2) it looks at the overall extent of sarcopenia, rather than just individual components, and includes sensitivity analyses that exclude individuals with probable sarcopenia, reinforcing the validity of findings related to confirmed sarcopenia; (3) it allows for more generalized conclusions by excluding individuals with dementia, as both biomarkers and performance tests could be confounded in this group; (4) it provides the longest follow‐up period currently available in the literature on this topic, offering a more comprehensive perspective on the progression of sarcopenia over time. Ladang's study provides relevant insights, having examined sarcopenia in a sample of 534 community‐dwelling older adults over an ongoing 10‐year follow‐up, despite only 16 subjects developing sarcopenia [12]. Our findings are consistent with those of this study, indicating that higher NfL levels are associated with an accelerated progression of sarcopenia in both sexes, particularly in individuals over 78 years of age. The observed association between NfL and incident sarcopenia, as well as between the Aβ ratio and incident sarcopenia in older participants, aligns with previous findings linking frailty to elevated NfL levels and decreased Aβ ratios, even in cognitively intact individuals. Notably, sarcopenia is considered a major contributor to physical frailty, suggesting that the relationship between AD biomarkers and frailty may be mediated by sarcopenia itself [33, 34]. Another aspect to consider in the interpretation of our results is that elevated NfL levels may indicate compromised neuromuscular junction health, leading to muscle fibre denervation and subsequent muscle atrophy, which are key features of sarcopenia [35]. This instability is particularly pronounced in older adults, where the combined effects of ageing and physical inactivity can exacerbate muscle loss [35]. Therefore, higher NfL levels are likely associated with the deterioration of muscle function as we age. In our study, p‐tau181 did not maintain a significant association with sarcopenia incidence after adjusting for covariates, in contrast to NfL. P‐Tau181 is a biomarker more specifically associated with AD pathology and may not directly reflect the pathological processes influencing sarcopenia. On the other hand, NfL is a better indicator of systemic inflammation, neuronal damage and neurodegeneration [36]. A substantial body of literature has shown that inflammatory cytokines activate several molecular pathways involved in skeletal muscle wasting, leading to an imbalance between protein synthesis and catabolism. High levels of inflammatory cytokines have been demonstrated to be negatively correlated with muscle strength and mass [37], suggesting that elevated NfL levels may represent an ongoing pathological process with a more direct impact on muscle health compared with p‐tau181.

Consistent with previous research [38, 39, 40], we examined potential sex differences in the relationship between NfL levels and the progression of sarcopenia; however, associations were observed in both sexes. Previous studies have reported a negative correlation between plasma NfL levels and gait speed in females [40], suggesting that females may experience distinct patterns of muscle loss and strength decline compared with males. Hormonal changes, particularly during menopause, could further influence muscle mass and strength [13, 35]. This may render females more susceptible to the effects of high NfL levels on sarcopenia, as evidenced by the fact that associations with sarcopenia incidence were predominantly observed in this subgroup. In contrast, the correlation between NfL levels and sarcopenia progression in males may be explained by two possible factors. First, men generally have a higher muscle mass than women, and as this decreases with age, especially in the presence of neurodegeneration or neuroinflammation, the impact of elevated NfL levels on muscle function may be more pronounced. Furthermore, neuroinflammation, mediated by pro‐inflammatory cytokines such as TNF‐α and IL‐6, is known to contribute to muscle atrophy. As gender is an important determinant of some types of muscle inflammation or a more pronounced decline in neuromuscular function [41], elevated NfL levels may be more strongly associated with sarcopenia progression in this group.

In our study, elevated levels of p‐tau181 were significantly associated with an increased risk of early progression of sarcopenia, particularly in males and participants over the age of 78 years. Regarding incident sarcopenia, this biomarker was significantly linked to a higher risk in males and younger individuals (under 78 years). To our knowledge, this is the first investigation to explore the relationship between p‐tau181 and sarcopenia in a large sample of older adults, as existing literature has primarily focused on animal models or post‐mortem analyses [14]. Mouse model studies, for instance, have suggested mitochondrial dysfunction as a potential underlying mechanism of this ‘muscle–brain axis’. Mitochondrial function declines with age, and in some individuals, it falls below a critical threshold, initiating a cascade of events that culminates in a pathology resembling AD, including neurodegeneration and the accumulation of tau, amyloid precursor protein and Aβ [42]. In these models, mitochondrial dysfunction has been shown to lead to increased amyloid plaque formation, inhibition of respiratory complex IV, resulting in tau deposition in the frontal cortex, and the administration of complex I inhibitors that cause a redistribution of tau from axons to cell bodies. However, human data on this phenomenon remain sparse. Given the role of p‐tau181 as a biomarker for AD [37], associated with amyloid plaques and neurofibrillary tangles [37], these findings may reflect a broader neurobiological vulnerability contributing to sarcopenia development, beyond typical age‐related processes. It is possible that, in young males, higher levels of testosterone may influence the production and metabolism of tau protein, leading to a greater accumulation of this biomarker compared with females at this age. This accumulation could, in turn, impair muscle protein synthesis. In females, the sharp decline in oestrogen levels after menopause contrasts with the gradual reduction of testosterone in males, potentially contributing to sex‐specific patterns of p‐tau181 accumulation and related neurodegenerative processes [38]. However, these remain speculative, and more research is needed to confirm these potential mechanisms.

Our study has several limitations. We used a modified version of the EWGSOP2 criteria based on calf circumference for muscle mass assessment, a method that, while practical, is less accurate compared with more advanced techniques such as bioelectrical impedance analysis and dual‐energy X‐ray absorptiometry. Additionally, the small number of participants with mild/moderate sarcopenia prevented us from analysing sarcopenia stages separately. Moreover, AD biomarkers were measured in serum, where concentrations are generally lower than in plasma, although serum and plasma biomarkers have shown strong correlation and high diagnostic accuracy for dementia prediction [39]. Furthermore, the SNAC‐K cohort consists of relatively affluent and generally healthy participants from a central district in Stockholm (Sweden), potentially affecting the generalizability of our results to other populations. Despite these limitations, our study has notable strengths. We analysed a large population sample and measured five different blood biomarkers of AD. Additionally, the long follow‐up period with frequent assessments (especially for participants ≥ 78 years), the use of a standardized definition of sarcopenia and the advanced statistical analyses to estimate sarcopenia latent profiles enhance the robustness and novelty of our findings. By excluding individuals with dementia, we aimed to ensure that the observed relationships between sarcopenia and biomarkers were not confounded by the cognitive decline associated with dementia, given the elevated levels of neurodegeneration biomarkers in this condition and the significant impact of dementia on physical function.

In conclusion, our study suggests that biomarkers such as p‐tau181 and NfL could be valuable biomarkers for the early identification of sarcopenia, particularly in older adults and men. Our results also support the potential involvement of AD pathology in the onset and progression of sarcopenia. However, further research is needed to determine the predictive value of these biomarkers for sarcopenia and to gain a deeper understanding of the pathophysiological mechanisms linking brain function and muscle health. These biomarkers could serve as valuable tools to more accurately identify patients at risk of sarcopenia, allowing for personalized interventions aimed at preserving functional autonomy and delaying the loss of independence as much as possible. Furthermore, future research could focus on establishing specific cut‐offs that clinicians could use to more precisely identify patients at risk of sarcopenia, thereby facilitating the implementation of targeted treatments to mitigate the impact of sarcopenia on quality of life and overall health.

## Author Contributions

Conceptualization: Davide Liborio Vetrano, Caterina Gregorio and Chiara Ceolin. Methodology: Caterina Gregorio, Chiara Ceolin and Alice Margherita Ornago. Formal Analysis: Caterina Gregorio and Chiara Ceolin. Writing the original draft: Chiara Ceolin. Writing review and rditing: Chiara Ceolin, Caterina Gregorio, Alice Margherita Ornago, Giulia Grande, Martina Valletta, Caterina Trevisan, Adrián Carballo Casla, Giuseppe Sergi, Amaia Calderón‐Larrañaga and Davide Liborio Vetrano. Visualization: Chiara Ceolin and Caterina Gregorio. Supervision: Davide Liborio Vetrano and Giuseppe Sergi.

## Conflicts of Interest

The authors declare no conflicts of interest.

## Supporting information


**Figure S1** Study population flow‐chart.
**Table S1. Multivariate logistic regression examining potential biomarkers associated with early sarcopenia progression, stratified by age (≥78 and <78 years).** *Notes:* Model 1 is adjusted for sex, age, and education; Model 2 is additionally adjusted for smoking and alcohol consumption, physical activity, as well as comorbidities including diabetes, heart, cerebrovascular, and chronic kidney disease.
**Table S2. Multivariate logistic regression examining potential biomarkers associated with the early sarcopenia progression, stratified by sex.** *Notes*: Model 1 is adjusted for age and education; Model 2 is additionally adjusted for smoking and alcohol habits, as well as comorbidities including diabetes, heart, cerebrovascular diseases, and chronic kidney disease, and physical activity.
**Table S3. Multivariate logistic regression examining potential biomarkers associated with the early sarcopenia progression, in people with dietary information, all sample (*n* = 1908).** *Notes*: Model 1 is adjusted for age and education; Model 2 is additionally adjusted for smoking and alcohol habits, as well as comorbidities including diabetes, heart, cerebrovascular diseases, and chronic kidney disease, and physical activity; Model 3 is additionally adjusted for dietary variables (energy intake, protein intake, and adherence to Mediterranean diet).
**Table S4. Multivariate logistic regression examining potential biomarkers associated with the early sarcopenia progression, in people with dietary information, by sex.** *Notes*: Model 1 is adjusted for age and education; Model 2 is additionally adjusted for smoking and alcohol habits, as well as comorbidities including diabetes, heart, cerebrovascular diseases, and chronic kidney disease, and physical activity; Model 3 is additionally adjusted for dietary variables (energy intake, protein intake, and adherence to Mediterranean diet).
**Table S5. Multivariate logistic regression examining potential biomarkers associated with the early sarcopenia progression, in people with dietary information, by age (≥78 and <78 years).** *Notes*: Model 1 is adjusted for sex and education; Model 2 is additionally adjusted for smoking and alcohol habits, as well as comorbidities including diabetes, heart, cerebrovascular diseases, and chronic kidney disease, and physical activity; Model 3 is additionally adjusted for dietary variables (energy intake, protein intake, and adherence to Mediterranean diet).
**Table S6. Cox regression analyses examining the independent association between biomarkers and sarcopenia incidence, in all sample and stratified by age (≥78 and <78 years).** *Notes*: Model 1 is adjusted for sex and education; Model 2 is additionally adjusted for smoking and alcohol habits, as well as comorbidities including diabetes, heart, cerebrovascular, and chronic kidney disease, and physical activity.
**Table S7. Cox regression analyses examining the independent association between biomarkers and sarcopenia incidence, in all sample and stratified by sex.** *Notes*: Model 1 is adjusted for age and education; Model 2 is additionally adjusted for smoking and alcohol habits, as well as comorbidities including diabetes, heart, cerebrovascular, and chronic kidney disease, and physical activity.
**Table S8. Cox regression sensitivity analyses examining the independent association between biomarkers and sarcopenia incidence (excluding people with probable sarcopenia at baseline), all sample (*n* = 1608).** *Notes*: Model 1 is adjusted for sex, age and education; Model 2 is additionally adjusted for smoking and alcohol habits, as well as comorbidities including diabetes, heart, cerebrovascular, and chronic kidney disease, and physical activity.
**Table S9. Cox regression sensitivity analyses examining the independent association between biomarkers and sarcopenia incidence (excluding people with probable sarcopenia at baseline), stratified by age (≥78 and <78 years).** *Analyses were conducted on a sample of 1608 participants. No. cases of incident sarcopenia: 115. Notes*: Model 1 is adjusted for sex and education; Model 2 is additionally adjusted for smoking and alcohol habits, as well as comorbidities including diabetes, heart, cerebrovascular, and chronic kidney disease, and physical activity.
**Table S10. Cox regression sensitivity analyses examining the independent association between biomarkers and sarcopenia incidence (excluding people with probable sarcopenia at baseline), stratified by sex.** *Analyses were conducted on a sample of 1608 participants. No. cases of incident sarcopenia: 115. Notes*: Model 1 is adjusted for age and education; Model 2 is additionally adjusted for smoking and alcohol habits, as well as comorbidities including diabetes, heart, cerebrovascular, and chronic kidney disease, and physical activity.
**Table S11. Cox regression sensitivity analyses examining the independent association between biomarkers and sarcopenia incidence (excluding people with musculoskeletal disorders at baseline), all sample (*n* = 1343).** *Notes*: Model 1 is adjusted for sex, age and education; Model 2 is additionally adjusted for smoking and alcohol habits, as well as comorbidities including diabetes, heart, cerebrovascular, and chronic kidney disease, and physical activity.
**Table S12. Cox regression sensitivity analyses examining the independent association between biomarkers and sarcopenia incidence (excluding people with musculoskeletal disorders at baseline), stratified by age (≥78 and <78 years).** *Analyses were conducted on a sample of 1343 participants. No. cases of incident sarcopenia: 97. Notes*: Model 1 is adjusted for sex and education; Model 2 is additionally adjusted for smoking and alcohol habits, as well as comorbidities including diabetes, heart, cerebrovascular, and chronic kidney disease, and physical activity.
**Table S13. Cox regression sensitivity analyses examining the independent association between biomarkers and sarcopenia incidence (excluding people with musculoskeletal disorders at baseline), stratified by sex.** *Analyses were conducted on a sample of 1343 participants. No. cases of incident sarcopenia: 97. Notes*: Model 1 is adjusted for age and education; Model 2 is additionally adjusted for smoking and alcohol habits, as well as comorbidities including diabetes, heart, cerebrovascular, and chronic kidney disease, and physical activity.
**Table S14. Cox regression sensitivity analyses examining the independent association between biomarkers and sarcopenia incidence, in people with dietary information, all sample (*n* = 1908).** *Notes*: Model 1 is adjusted for sex, age and education; Model 2 is additionally adjusted for smoking and alcohol habits, as well as comorbidities including diabetes, heart, cerebrovascular diseases, and chronic kidney disease, and physical activity; Model 3 is additionally adjusted for dietary variables (energy intake, protein intake, and adherence to Mediterranean diet).
**Table S15. Cox regression sensitivity analyses examining the independent association between biomarkers and sarcopenia incidence, in people with dietary information, by sex.** *Notes*: Model 1 is adjusted for age and education; Model 2 is additionally adjusted for smoking and alcohol habits, as well as comorbidities including diabetes, heart, cerebrovascular diseases, and chronic kidney disease, and physical activity; Model 3 is additionally adjusted for dietary variables (energy intake, protein intake, and adherence to Mediterranean diet).
**Table S16. Cox regression sensitivity analyses examining the independent association between biomarkers and sarcopenia incidence, in people with dietary information, by age (≥78 and <78 years).** *Notes*: Model 1 is adjusted for sex and education; Model 2 is additionally adjusted for smoking and alcohol habits, as well as comorbidities including diabetes, heart, cerebrovascular diseases, and chronic kidney disease, and physical activity; Model 3 is additionally adjusted for dietary variables (energy intake, protein intake, and adherence to Mediterranean diet).
