# Supplementary material for: Association of Alzheimer's Disease Blood Biomarkers With Sarcopenia Incidence and Progression: A 12‐Year Population‐Based Study
Source: J Cachexia Sarcopenia Muscle. 2025 Jun 9;16(3):e13835. doi: 10.1002/jcsm.13835 (PMC12148950; doi:10.1002/jcsm.13835)
Supplement: Supplementary file 1 — Figure S1 Study population flow‐chart. Table S1. Multivariate logistic regression examining potential biomarkers associated with early sarcopenia progression, stratified by age (≥78 and <78 years). Notes: Model 1 is adjusted for sex, age, and education; Model 2 is additionally adjusted for smoking and alcohol consumption, physical activity, as well as comorbidities including diabetes, heart, cerebrovascular, and chronic kidney disease. Table S2. Multivariate logistic regression examining potential biomarkers associated with the early sarcopenia progression, stratified by sex. Notes: Model 1 is adjusted for age and education; Model 2 is additionally adjusted for smoking and alcohol habits, as well as comorbidities including diabetes, heart, cerebrovascular diseases, and chronic kidney disease, and physical activity. Table S3. Multivariate logistic regression examining potential biomarkers associated with the early sarcopenia progression, in people with dietary information, all sample (n = 1908). Notes: Model 1 is adjusted for age and education; Model 2 is additionally adjusted for smoking and alcohol habits, as well as comorbidities including diabetes, heart, cerebrovascular diseases, and chronic kidney disease, and physical activity; Model 3 is additionally adjusted for dietary variables (energy intake, protein intake, and adherence to Mediterranean diet). Table S4. Multivariate logistic regression examining potential biomarkers associated with the early sarcopenia progression, in people with dietary information, by sex. Notes: Model 1 is adjusted for age and education; Model 2 is additionally adjusted for smoking and alcohol habits, as well as comorbidities including diabetes, heart, cerebrovascular diseases, and chronic kidney disease, and physical activity; Model 3 is additionally adjusted for dietary variables (energy intake, protein intake, and adherence to Mediterranean diet). Table S5. Multivariate logistic regression examining potential biomarkers associated [file JCSM-16-e13835-s001.docx]

**Supplementary Figure 1. Study population flow-chart.**

3363 SNAC-K participants ≥ 60 years

**341** participants excluded:

- 240 with dementia diagnosis

- 40 with Parkinson’s disease or parkinsonism

- 4 with multiple sclerosis

- 191 living in institutions

3022 participants

**40** participants excluded:

- with missing sarcopenia

**2291** participants ≥ 60 years

2982 participants

**691** participants excluded:

- with at least one biomarker missing

**Supplementary Material. List of all the 60 disease categories considered in the study.**

| Hypertension | Other cardiovascular disease |
| --- | --- |
| Dyslipidemia | Neurotic, stress-related and somatoform diseases |
| Chronic kidney diseases | [Other genitourinary diseases](https://pmc.ncbi.nlm.nih.gov/articles/PMC5861938/table/T1/#fn-02) |
| Ischemic heart disease | Cardiac valve diseases |
| Anemia | Migraine and facial pain syndromes |
| Osteoarthritis and other degenerative joint diseases | [Other psychiatric and behavioral diseases](https://pmc.ncbi.nlm.nih.gov/articles/PMC5861938/table/T1/#fn-02) |
| Colitis and related diseases | [Other neurological diseases](https://pmc.ncbi.nlm.nih.gov/articles/PMC5861938/table/T1/#fn-02) |
| Deafness, hearing impairment | Sleep disorders |
| Heart failure | Bradycardias and conduction diseases |
| Obesity | Peripheral vascular disease |
| Thyroid diseases | [Other metabolic diseases](https://pmc.ncbi.nlm.nih.gov/articles/PMC5861938/table/T1/#fn-02) |
| Dementia | Peripheral neuropathy |
| Atrial fibrillation | Chronic pancreas, biliary tract and gallbladder diseases |
| Depression and mood diseases | Allergy |
| Solid neoplasms | Parkinson and parkinsonism |
| Diabetes | [Other respiratory diseases](https://pmc.ncbi.nlm.nih.gov/articles/PMC5861938/table/T1/#fn-02) |
| Cerebrovascular disease | Chronic ulcer of the skin |
| Osteoporosis | Epilepsy |
| [Other musculoskeletal and joint diseases](https://pmc.ncbi.nlm.nih.gov/articles/PMC5861938/table/T1/#fn-02) | Ear, nose, throat diseases |
| Dorsopathies | Inflammatory bowel diseases |
| Glaucoma | Hematological neoplasms |
| Cataract and other lens diseases | Venous and lymphatic diseases |
| Asthma | Schizophrenia and delusional diseases |
| [Other eye diseases](https://pmc.ncbi.nlm.nih.gov/articles/PMC5861938/table/T1/#fn-02) | Blood and blood forming organ diseases |
| COPD, emphysema, chronic bronchitis | [Other digestive diseases](https://pmc.ncbi.nlm.nih.gov/articles/PMC5861938/table/T1/#fn-02) |
| Autoimmune diseases | Chronic infectious diseases |
| Blindness, visual impairment | Chronic liver diseases |
| Esophagus, stomach, and duodenum diseases | Multiple sclerosis |
| Prostate diseases | [Other skin diseases](https://pmc.ncbi.nlm.nih.gov/articles/PMC5861938/table/T1/#fn-02) |
| Inflammatory arthropathies | Chromosomal abnormalities |

**Supplementary Table 1. Multivariate logistic regression examining potential biomarkers associated with early sarcopenia progression, stratified by age (≥78 and <78 years).**

|  | **Age <78 years (n=1424)** | | | | **Age ≥78 years (n=833)** | | | |
| --- | --- | --- | --- | --- | --- | --- | --- | --- |
|  | **Model 1** | | **Model 2** | | **Model 1** | | **Model 2** | |
|  | **OR (95% CI)** | **p-value** | **OR (95% CI)** | **p-value** | **OR (95% CI)** | **p-value** | **OR (95% CI)** | **p-value** |
| ***Aβ42/40*** | 1.06 (0.97;1.18) | 0.20 | 1.07 (0.96;1.19) | 0.20 | 0.84 (0.70;1.01) | 0.061 | 0.88 (0.73;1.05) | 0.20 |
| ***t-tau*** | 0.97 (0.86;1.10) | 0.70 | 0.95 (0.83;1.08) | 0.50 | **1.26 (1.08;1.49)** | **0.006** | 1.15 (0.99;1.37) | 0.10 |
| ***p-tau181*** | 1.20 (0.99;1.47) | 0.061 | 1.18 (0.97;1.44) | 0.11 | **1.43 (1.21;1.73)** | **<0.001** | **1.32 (1.11;1.59)** | **0.003** |
| ***NfL*** | **1.34 (1.07;1.77)** | **0.022** | 1.27 (0.92;1.68) | 0.067 | **1.87 (1.51;2.36)** | **<0.001** | **1.77 (1.40;2.28)** | **<0.001** |
| ***GFAP*** | 1.06 (0.95;1.23) | 0.40 | 1.06 (0.95;1.24) | 0.30 | 1.04 (0.91;1.25) | 0.60 | 1.00 (0.87;1.19) | 0.90 |

*Notes:* Model 1 is adjusted for sex, age, and education; Model 2 is additionally adjusted for smoking and alcohol consumption, physical activity, as well as comorbidities including diabetes, heart, cerebrovascular, and chronic kidney disease.

**Supplementary Table 2. Multivariate logistic regression examining potential biomarkers associated with the early sarcopenia progression, stratified by sex.**

|  | **Male (n=882)** | | | | **Female (n=1409)** | | | |
| --- | --- | --- | --- | --- | --- | --- | --- | --- |
|  | **Model 1** | | **Model 2** | | **Model 1** | | **Model 2** | |
|  | **OR (95% CI)** | **p-value** | **OR (95% CI)** | **p-value** | **OR (95% CI)** | **p-value** | **OR (95% CI)** | **p-value** |
| ***Aβ42/40*** | 0.97 (0.84;1.12) | 0.70 | 0.96 (0.83;1.12) | 0.60 | 1.05 (0.94;1.17) | 0.40 | 1.05 (0.94;1.18) | 0.40 |
| ***t-tau*** | 1.07 (0.95;1.23) | 0.30 | 1.02 (0.91;1.17) | 0.70 | 1.07 (0.93;1.22) | 0.40 | 1.04 (0.91;1.20) | 0.60 |
| ***p-tau181*** | **1.45 (1.20;1.80)** | **<0.001** | **1.39 (1.14;1.73)** | **0.002** | 1.18 (1.00;1.42) | 0.069 | 1.13 (0.96;1.36) | 0.20 |
| ***NfL*** | **1.55 (1.22;2.05)** | **0.001** | **1.38 (1.11;1.82)** | **0.012** | **1.77 (1.39;2.31)** | **<0.001** | **1.75 (1.35;2.32)** | **<0.001** |
| ***GFAP*** | 1.05 (0.95;1.18) | 0.40 | 1.05 (0.95;1.19) | 0.40 | 1.05 (0.85;1.32) | 0.60 | 1.04 (0.83;1.30) | 0.70 |

*Notes*: Model 1 is adjusted for age and education; Model 2 is additionally adjusted for smoking and alcohol habits, as well as comorbidities including diabetes, heart, cerebrovascular diseases, and chronic kidney disease, and physical activity.

**Supplementary Table 3. Multivariate logistic regression examining potential biomarkers associated with the early sarcopenia progression, in people with dietary information, all sample (n=1908).**

|  | **Model 1** | | **Model 2** | | **Model 3** | |
| --- | --- | --- | --- | --- | --- | --- |
|  | **OR (95% CI)** | **p-value** | **OR (95% CI)** | **p-value** | **OR (95% CI)** | **p-value** |
| ***Aβ42/40*** | 1.05  (0.81;1.37) | 0.69 | 1.02  (0.93;1.11) | 0.72 | 1.02  (0.93;1.11) | 0.72 |
| ***t-tau*** | 1.23  (0.86;1.47) | 0.39 | 1.03  (0.93;1.13) | 0.59 | 1.03  (0.93;1.13) | 0.55 |
| ***p-tau181*** | 1.28  (1.01;1.03) | **0.002** | **1.23**  **(1.06;1.43)** | **0.008** | **1.30**  **(1.06;1.43)** | **0.007** |
| ***NfL*** | **1.87**  **(1.23;2.83)** | **0.003** | **1.45**  **(1.17;1.80)** | **0.001** | **1.45**  **(1.17;1.80)** | **0.001** |
| ***GFAP*** | 0.84  (0.55;1.29) | 0.43 | 1.06  (0.96;1.16) | 0.26 | 1.06  (0.96;1.16) | 0.26 |

*Notes*: Model 1 is adjusted for age and education; Model 2 is additionally adjusted for smoking and alcohol habits, as well as comorbidities including diabetes, heart, cerebrovascular diseases, and chronic kidney disease, and physical activity; Model 3 is additionally adjusted for dietary variables (energy intake, protein intake, and adherence to Mediterranean diet).

**Supplementary Table 4. Multivariate logistic regression examining potential biomarkers associated with the early sarcopenia progression, in people with dietary information, by sex.**

|  | **Male (n=757)** | | | | | | **Female (n=1151)** | | | | | |
| --- | --- | --- | --- | --- | --- | --- | --- | --- | --- | --- | --- | --- |
|  | **Model 1** | | **Model 2** | | **Model 3** | | **Model 1** | | **Model 2** | | **Model 3** | |
|  | **OR**  **(95% CI)** | **p-value** | **OR**  **(95% CI)** | **p-value** | **OR**  **(95% CI)** | **p-value** | **OR**  **(95% CI)** | **p-value** | **OR**  **(95% CI)** | **p-value** | **OR**  **(95% CI)** | **p-value** |
| ***Aβ42/40*** | 0.94 (0.79;1.13) | 0.53 | 0.93 (0.78;1.12) | 0.46 | 0.93 (0.78;1.18) | 0.46 | 1.04 (0.93;1.16) | 0.46 | 1.04 (0.93;1.16) | 0.47 | 1.04 (0.93;1.16) | 0.47 |
| ***t-tau*** | 1.04 (0.92;1.17) | 0.51 | 1.01 (0.89;1.14) | 0.86 | 1.01 (0.89;1.14) | 0.85 | 1.08 (0.92;1.26) | 0.35 | 1.06 (0.90;1.24) | 0.48 | 1.06 (0.90;1.24) | 0.48 |
| ***p-tau181*** | **1.41 (1.13;1.77)** | **0.002** | **1.37 (1.09;1.72)** | **0.007** | **1.37 (1.09;1.73)** | **0.006** | 1.17 (0.95;1.46) | 0.14 | 1.13 (0.90;1.40) | 0.28 | 1.12 (0.90;1.40) | 0.29 |
| ***NfL*** | 1.25 (0.99;1.57) | 0.05 | 1.19 (0.96;1.47) | 0.11 | 1.19 (0.96;1.48) | 0.11 | **1.96 (1.41;2.73)** | **<0.001** | **2.03 (1.43;2.89)** | **<0.001** | **2.03 (1.43;2.89)** | **<0.001** |
| ***GFAP*** | 1.04 (0.94;1.15) | 0.42 | 1.05 (0.95;1.15) | 0.38 | 1.05 (0.95;1.15) | 0.38 | 1.13 (0.87;1.47) | 0.34 | 1.14 (0.89;1.47) | 0.33 | 1.14 (0.88;1.47) | 0.33 |

*Notes*: Model 1 is adjusted for age and education; Model 2 is additionally adjusted for smoking and alcohol habits, as well as comorbidities including diabetes, heart, cerebrovascular diseases, and chronic kidney disease, and physical activity; Model 3 is additionally adjusted for dietary variables (energy intake, protein intake, and adherence to Mediterranean diet).

**Supplementary Table 5. Multivariate logistic regression examining potential biomarkers associated with the early sarcopenia progression, in people with dietary information, by age (≥78 and <78 years).**

|  | **Age <78 years (n=1304)** | | | | | | **Age ≥78 years (n=604)** | | | | | |
| --- | --- | --- | --- | --- | --- | --- | --- | --- | --- | --- | --- | --- |
|  | **Model 1** | | **Model 2** | | **Model 3** | | **Model 1** | | **Model 2** | | **Model 3** | |
|  | **OR**  **(95% CI)** | **p-value** | **OR**  **(95% CI)** | **p-value** | **OR**  **(95% CI)** | **p-value** | **OR**  **(95% CI)** | **p-value** | **OR**  **(95% CI)** | **p-value** | **OR**  **(95% CI)** | **p-value** |
| ***Aβ42/40*** | 1.06 (0.95;1.17) | 0.31 | 1.06 (0.95;1.81) | 0.31 | 1.06 (0.95;1.18) | 0.31 | 0.84 (0.68;1.03) | 0.09 | 0.86 (0.70;1.06) | 0.15 | 0.86 (0.70;1.06) | 0.16 |
| ***t-tau*** | 0.96 (0.85;1.10) | 0.57 | 0.94 (0.82;1.08) | 0.39 | 0.94 (0.82;1.08) | 0.39 | **1.29 (1.05;1.59)** | **0.02** | 1.21 (0.97;1.50) | 0.09 | 1.22 (0.98;1.51) | 0.08 |
| ***p-tau181*** | 1.20 (0.98;1.47) | 0.08 | 1.16 (0.95;1.43) | 0.15 | 1.16 (0.95;1.43) | 0.15 | **1.44 (1.15;1.81)** | **0.002** | **1.37 (1.09;1.73)** | **0.007** | **1.38 (1.09;1.75)** | **0.006** |
| ***NfL*** | 1.27 (0.99;1.62) | 0.06 | 1.20 (0.95;1.52) | 0.12 | 1.20 (0.95;1.52) | 0.12 | **1.91 (1.42;2.57)** | **<0.001** | **2.01 (1.45;2.79)** | **<0.001** | **2.03 (1.46;2.82)** | **<0.001** |
| ***GFAP*** | 1.05 (0.93;1.19) | 0.40 | 1.06 (0.94;1.19) | 0.35 | 1.06 (0.94;1.19) | 0.35 | 1.06 (0.90;1.24) | 0.49 | 1.04 (0.88;1.23) | 0.62 | 1.04 (0.88;1.23) | 0.63 |

*Notes*: Model 1 is adjusted for sex and education; Model 2 is additionally adjusted for smoking and alcohol habits, as well as comorbidities including diabetes, heart, cerebrovascular diseases, and chronic kidney disease, and physical activity; Model 3 is additionally adjusted for dietary variables (energy intake, protein intake, and adherence to Mediterranean diet).

**Supplementary Table 6. Cox regression analyses examining the independent association between biomarkers and sarcopenia incidence, in all sample and stratified by age (≥78 and <78 years).**

|  | **Age <78 years (n=1424)** | | | | **Age ≥78 years (n=833)** | | | |
| --- | --- | --- | --- | --- | --- | --- | --- | --- |
|  | **Model 1** | | **Model 2** | | **Model 1** | | **Model 2** | |
|  | **HR 95% CI)** | **p-value** | **HR (95% CI)** | **p-value** | **HR (95% CI)** | **p-value** | **HR (95% CI)** | **p-value** |

| ***Aβ42/40*** | 1.01 (0.91;1.13) | 0.80 | 1.02 (0.93;1.13) | 0.60 | 0.95 (0.79;1.15) | 0.60 | 0.95 (0.79;1.14) | 0.50 |
| --- | --- | --- | --- | --- | --- | --- | --- | --- |
| ***t-tau*** | 1.00 (0.86;1.15) | 0.90 | 1.00 (0.86;1.16) | 0.90 | 0.90 (0.73;1.11) | 0.30 | 0.87 (0.70;1.09) | 0.20 |
| ***p-tau181*** | **1.35 (1.14;1.59)** | **<0.001** | **1.32 (1.11;1.58)** | **0.002** | **1.26 (1.01;1.59)** | **0.045** | 1.23 (0.97;1.57) | 0.090 |
| ***NfL*** | **1.15 (1.06;1.25)** | **<0.001** | **1.17 (1.07;1.27)** | **<0.001** | **1.36 (1.17;1.57)** | **<0.001** | **1.35 (1.15;1.58)** | **<0.001** |
| ***GFAP*** | 1.04 (0.95;1.14) | 0.40 | 1.05 (0.95;1.15) | 0.30 | 1.00 (0.83;1.21) | 0.90 | 1.00 (0.82;1.22) | 0.90 |

*Notes*: Model 1 is adjusted for sex and education; Model 2 is additionally adjusted for smoking and alcohol habits, as well as comorbidities including diabetes, heart, cerebrovascular, and chronic kidney disease, and physical activity.

**Supplementary Table 7. Cox regression analyses examining the independent association between biomarkers and sarcopenia incidence, in all sample and stratified by sex.**

|  | **Male (n=882)** | | | | **Female (n=1409)** | | | |
| --- | --- | --- | --- | --- | --- | --- | --- | --- |
|  | **Model 1** | | **Model 2** | | **Model 1** | | **Model 2** | |
|  | **HR (95% CI)** | **p-value** | **HR (95% CI)** | **p-value** | **HR (95% CI)** | **p-value** | **HR (95% CI)** | **p-value** |

| ***Aβ42/40*** | 1.08 (0.94;1.24) | 0.30 | 1.08 (0.94;1.24) | 0.30 | 1.05 (0.95;1.16) | 0.40 | 1.06 (0.96;1.17) | 0.20 |
| --- | --- | --- | --- | --- | --- | --- | --- | --- |
| ***t-tau*** | 1.0 (0.82;1.21) | 0.90 | 0.98 (0.80;1.20) | 0.80 | 0.89 (0.75;1.06) | 0.20 | 0.86 (0.72;1.03) | 0.10 |
| ***p-tau181*** | **1.38 (1.09;1.74)** | **0.007** | **1.40 (1.10;1.79)** | **0.006** | 1.06 (0.87;1.29) | 0.50 | 1.04 (0.85;1.28) | 0.70 |
| ***NfL*** | **1.15 (1.03;1.29)** | **0.015** | **1.16 (1.03;1.30)** | **0.013** | **1.43 (1.23;1.66)** | **<0.001** | **1.40 (1.20;1.63)** | **<0.001** |
| ***GFAP*** | 0.95 (0.76;1.19) | 0.60 | 0.97 (0.79;1.20) | 0.80 | 1.05 (0.84;1.30) | 0.70 | 1.02 (0.81;1.30) | 0.90 |

*Notes*: Model 1 is adjusted for age and education; Model 2 is additionally adjusted for smoking and alcohol habits, as well as comorbidities including diabetes, heart, cerebrovascular, and chronic kidney disease, and physical activity.

**Supplementary Table 8. Cox regression sensitivity analyses examining the independent association between biomarkers and sarcopenia incidence (excluding people with probable sarcopenia at baseline), all sample (n=1608).**

|  | **Model 1** | | **Model 2** | |
| --- | --- | --- | --- | --- |
|  | **HR (95% CI)** | **p-value** | **HR (95% CI)** | **p-value** |
| ***Aβ42/40*** | 1.04  (0.86;1.25) | 0.70 | 1.03  (0.85;1.25) | 0.80 |
| ***t-tau*** | 0.81  (0.61;1.07) | 0.13 | 0.79  (0.60;1.05) | 0.10 |
| ***p-tau181*** | 1.17  (0.91;1.51) | 0.20 | 1.24  (0.95;1.60) | 0.11 |
| ***NfL*** | 1.15  (1.00;1.32) | 0.058 | 1.13  (0.98;1.31) | 0.088 |
| ***GFAP*** | 1.09  (0.94;1.27) | 0.30 | 1.09  (0.95;1.25) | 0.20 |

*Notes*: Model 1 is adjusted for sex, age and education; Model 2 is additionally adjusted for smoking and alcohol habits, as well as comorbidities including diabetes, heart, cerebrovascular, and chronic kidney disease, and physical activity.

**Supplementary Table 9. Cox regression sensitivity analyses examining the independent association between biomarkers and sarcopenia incidence (excluding people with probable sarcopenia at baseline), stratified by age (≥78 and <78 years).**

|  | **Age <78 years (n=1270)** | | | | **Age ≥78 years (n=338)** | | | |
| --- | --- | --- | --- | --- | --- | --- | --- | --- |
|  | **Model 1** | | **Model 2** | | **Model 1** | | **Model 2** | |
|  | **HR**  **(95% CI)** | **p-value** | **HR**  **(95% CI)** | **p-value** | **HR**  **(95% CI)** | **p-value** | **HR**  **(95% CI)** | **p-value** |
| ***Aβ42/40*** | 1.08 (0.86;1.36) | 0.50 | 1.06 (0.83;1.35) | 0.60 | 0.82 (0.58;1.16) | 0.30 | 0.84 (0.59;1.18) | 0.30 |
| ***t-tau*** | 0.92 (0.62;1.37) | 0.70 | 0.97 (0.67;1.39) | 0.90 | 0.81 (0.57;1.16) | 0.20 | 0.76 (0.53;1.08) | 0.13 |
| ***p-tau181*** | 1.27 (0.85;1.91) | 0.20 | 1.36 (0.91;2.02) | 0.13 | **1.46 (1.06;2.01)** | **0.019** | **1.52 (1.09;2.14)** | **0.015** |
| ***NfL*** | 1.14 (0.94;1.37) | 0.20 | 1.14 (0.94;1.38) | 0.20 | 1.12 (0.96;1.30) | 0.20 | 1.14 (0.95;1.36) | 0.15 |
| ***GFAP*** | 1.01 (0.80;1.28) | 0.90 | 1.02 (0.81;1.27) | 0.90 | 1.19 (0.95;1.51) | 0.14 | **1.30 (1.02;1.66)** | **0.037** |

*Analyses were conducted on a sample of 1608 participants. No. cases of incident sarcopenia: 115.*

*Notes*: Model 1 is adjusted for sex and education; Model 2 is additionally adjusted for smoking and alcohol habits, as well as comorbidities including diabetes, heart, cerebrovascular, and chronic kidney disease, and physical activity.

**Supplementary Table 10. Cox regression sensitivity analyses examining the independent association between biomarkers and sarcopenia incidence (excluding people with probable sarcopenia at baseline), stratified by sex.**

|  | **Male (n=704)** | | | | **Female (n=904)** | | | |
| --- | --- | --- | --- | --- | --- | --- | --- | --- |
|  | **Model 1** | | **Model 2** | | **Model 1** | | **Model 2** | |
|  | **HR**  **(95% CI)** | **p-value** | **HR**  **(95% CI)** | **p-value** | **HR**  **(95% CI)** | **p-value** | **HR**  **(95% CI)** | **p-value** |
| ***Aβ42/40*** | 1.08 (0.85;1.38) | 0.50 | 1.11 (0.87;1.42) | 0.40 | 0.99 (0.75;1.31) | 0.90 | 0.91 (0.67;1.26) | 0.60 |
| ***t-tau*** | 0.99 (0.67;1.47) | 0.90 | 0.95 (0.63;1.45) | 0.80 | **0.67 (0.46;0.99)** | **0.044** | 0.70 (0.48;1.04) | 0.078 |
| ***p-tau181*** | 1.36 (0.96;1.92) | 0.087 | **1.50 (1.04;2.18)** | **0.032** | 1.02 (0.70;1.48) | 0.90 | 1.07 (0.74;1.56) | 0.70 |
| ***NfL*** | 1.16 (0.90;1.49) | 0.30 | 1.18 (0.93;1.49) | 0.20 | 1.15 (0.97;1.37) | 0.12 | 1.19 (0.96;1.49) | 0.11 |
| ***GFAP*** | 1.09 (0.92;1.30) | 0.30 | 1.11 (0.95;1.29) | 0.20 | 1.06 (0.70;1.62) | 0.80 | 1.11 (0.70;1.75) | 0.70 |

*Analyses were conducted on a sample of 1608 participants. No. cases of incident sarcopenia: 115.*

*Notes*: Model 1 is adjusted for age and education; Model 2 is additionally adjusted for smoking and alcohol habits, as well as comorbidities including diabetes, heart, cerebrovascular, and chronic kidney disease, and physical activity.

**Supplementary Table 11. Cox regression sensitivity analyses examining the independent association between biomarkers and sarcopenia incidence (excluding people with musculoskeletal disorders at baseline), all sample (n=1343).**

|  | **Model 1** | | **Model 2** | |
| --- | --- | --- | --- | --- |
|  | **HR (95% CI)** | **p-value** | **HR (95% CI)** | **p-value** |
| ***Aβ42/40*** | 1.07  (0.98;1-16) | 0.15 | 1.06  (0.97;1.16) | 0.20 |
| ***t-tau*** | 0.94  (0.81;1.08) | 0.40 | 0.93  (0.81;1.08) | 0.40 |
| ***p-tau181*** | **1.17**  **(1.00;1.37)** | **0.047** | 1.15  (0.98;1.35) | 0.10 |
| ***NfL*** | **1.19**  **(1.08;1.30)** | **<0.001** | **1.19**  **(1.08;1.31)** | **<0.001** |
| ***GFAP*** | 1.04  (0.93;1.16) | 0.50 | 1.05  (0.94;1.17) | 0.40 |

*Notes*: Model 1 is adjusted for sex, age and education; Model 2 is additionally adjusted for smoking and alcohol habits, as well as comorbidities including diabetes, heart, cerebrovascular, and chronic kidney disease, and physical activity.

**Supplementary Table 12. Cox regression sensitivity analyses examining the independent association between biomarkers and sarcopenia incidence (excluding people with musculoskeletal disorders at baseline), stratified by age (≥78 and <78 years).**

|  | **Age <78 years (n=1076)** | | | | **Age ≥78 years (n=267)** | | | |
| --- | --- | --- | --- | --- | --- | --- | --- | --- |
|  | **Model 1** | | **Model 2** | | **Model 1** | | **Model 2** | |
|  | **HR**  **(95% CI)** | **p-value** | **HR**  **(95% CI)** | **p-value** | **HR**  **(95% CI)** | **p-value** | **HR**  **(95% CI)** | **p-value** |
| ***Aβ42/40*** | 1.03 (0.93;1.15) | 0.60 | 1.04 (0.94;1.15) | 0.50 | 0.91 (0.73;1.13) | 0.40 | 0.86 (0.68;1.08) | 0.20 |
| ***t-tau*** | 0.96 (0.80;1.14) | 0.60 | 0.94 (0.79;1.13) | 0.50 | 0.90 (0.72;1-12) | 0.30 | 0.90 (0.71;1-14) | 0.40 |
| ***p-tau181*** | **1.32 (1.11;1.58)** | **0.002** | **1.30 (1.07;1.57)** | **0.007** | **1.34 (1.03;1.74)** | **0.028** | 1.27 (0.97;1.66) | 0.084 |
| ***NfL*** | **1.12 (1.01;1.24)** | **0.032** | **1.13 (1.01;1.26)** | **0.033** | **1.37 (1.17;1.61)** | **<0.001** | **1.34 (1.12;1.60)** | **0.001** |
| ***GFAP*** | 1.03 (0.94;1.14) | 0.50 | 1.04 (0.94;1.15) | 0.40 | 1.02 (0.83;1.25) | 0.80 | 1.03 (0.83;1.28) | 0.80 |

*Analyses were conducted on a sample of 1343 participants. No. cases of incident sarcopenia: 97.*

*Notes*: Model 1 is adjusted for sex and education; Model 2 is additionally adjusted for smoking and alcohol habits, as well as comorbidities including diabetes, heart, cerebrovascular, and chronic kidney disease, and physical activity.

**Supplementary Table 13. Cox regression sensitivity analyses examining the independent association between biomarkers and sarcopenia incidence (excluding people with musculoskeletal disorders at baseline), stratified by sex.**

|  | **Male (n=595)** | | | | **Female (n=748)** | | | |
| --- | --- | --- | --- | --- | --- | --- | --- | --- |
|  | **Model 1** | | **Model 2** | | **Model 1** | | **Model 2** | |
|  | **HR**  **(95% CI)** | **p-value** | **HR**  **(95% CI)** | **p-value** | **HR**  **(95% CI)** | **p-value** | **HR**  **(95% CI)** | **p-value** |
| ***Aβ42/40*** | 1.12 (0.99;1.27) | 0.074 | 1.10 (0.97;1.25) | 0.14 | 1.02 (0.90;1.16) | 0.80 | 1.02 (0.89;1.17) | 0.80 |
| ***t-tau*** | 0.99 (0.80;1.22) | 0.90 | 0.97 (0.77;1.21) | 0.80 | 0.90 (0.74;1.09) | 0.30 | 0.90 (0.74;1.10) | 0.30 |
| ***p-tau181*** | **1.48 (1.17;1.86)** | **<0.001** | **1.50 (1.18;1.92)** | **<0.001** | 1.04 (0.84;1.28) | 0.70 | 1.01 (0.81;1.27) | 0.90 |
| ***NfL*** | 1.12 (0.97;1.30) | 0.13 | 1.12 (0.96;1.31) | 0.15 | **1.45 (1.23;1.71)** | **<0.001** | **1.39 (1.16;1.66)** | **<0.001** |
| ***GFAP*** | 0.95 (0.75;1.20) | 0.70 | 0.98 (0.79;1.21) | 0.80 | **1.28 (1.02;1.61)** | **0.035** | 1.25 (1.00;1.56) | 0.050 |

*Analyses were conducted on a sample of 1343 participants. No. cases of incident sarcopenia: 97.*

*Notes*: Model 1 is adjusted for age and education; Model 2 is additionally adjusted for smoking and alcohol habits, as well as comorbidities including diabetes, heart, cerebrovascular, and chronic kidney disease, and physical activity.

**Supplementary Table 14. Cox regression sensitivity analyses examining the independent association between biomarkers and sarcopenia incidence, in people with dietary information, all sample (n=1908).**

|  | **Model 1** | | **Model 2** | | **Model 3** | |
| --- | --- | --- | --- | --- | --- | --- |
|  | **OR (95% CI)** | **p-value** | **OR (95% CI)** | **p-value** | **OR (95% CI)** | **p-value** |
| ***Aβ42/40*** | 0.91  (0.76;1.08) | 0.29 | 0.92  (0.77;1.09) | 0.33 | 0.92  (0.77;1.10) | 0.35 |
| ***t-tau*** | 0.96  (0.80;1.16) | 0.70 | 0.97  (0.80;1.17) | 0.75 | 0.97  (0.80;1.17) | 0.73 |
| ***p-tau181*** | **1.18**  **(1.01;1.41)** | **0.04** | **1.19**  **(1.01;1.41)** | **0.04** | **1.19**  **(1.01;1.41)** | **0.04** |
| ***NfL*** | **1.17**  **(1.05;1.30)** | **0.004** | **1.17**  **(1.05;1.30)** | **0.004** | **1.17**  **(1.05;1.30)** | **0.005** |
| ***GFAP*** | 1.07  (0.93;1.23) | 0.36 | 1.08  (0.95;1.22) | 0.30 | 1.08  (0.96;1.21) | 0.23 |

*Notes*: Model 1 is adjusted for sex, age and education; Model 2 is additionally adjusted for smoking and alcohol habits, as well as comorbidities including diabetes, heart, cerebrovascular diseases, and chronic kidney disease, and physical activity; Model 3 is additionally adjusted for dietary variables (energy intake, protein intake, and adherence to Mediterranean diet).

**Supplementary Table 15. Cox regression sensitivity analyses examining the independent association between biomarkers and sarcopenia incidence, in people with dietary information, by sex.**

|  | **Male (n=757)** | | | | | | **Female (n=1151)** | | | | | |
| --- | --- | --- | --- | --- | --- | --- | --- | --- | --- | --- | --- | --- |
|  | **Model 1** | | **Model 2** | | **Model 3** | | **Model 1** | | **Model 2** | | **Model 3** | |
|  | **OR**  **(95% CI)** | **p-value** | **OR**  **(95% CI)** | **p-value** | **OR**  **(95% CI)** | **p-value** | **OR**  **(95% CI)** | **p-value** | **OR**  **(95% CI)** | **p-value** | **OR**  **(95% CI)** | **p-value** |
| ***Aβ42/40*** | 0.94 (0.71;1.26) | 0.70 | 0.96 (0.73;1.27) | 0.80 | 0.98 (0.75;1.27) | 0.88 | 0.90 (0.72;1.13) | 0.36 | 0.89 (0.71;1.13) | 0.36 | 0.91 (0.71;1.15) | 0.41 |
| ***t-tau*** | 0.93 (0.66;1.32) | 0.70 | 0.90 (0.62;1.31) | 0.59 | 0.93 (0.64;1.35) | 0.72 | 0.97 (0.77;1.21) | 0.78 | 1.00 (0.80;1.26) | 0.98 | 0.99 (0.78;1.24) | 0.93 |
| ***p-tau181*** | 1.22 (0.97;1.54) | 0.09 | 1.16 (0.90;1.50) | 0.24 | 1.18 (0.93;1.50) | 0.18 | 1.12 (0.87;1.43) | 0.39 | 1.17 (0.92;1.50) | 0.21 | 1.16 (0.90;1.49) | 0.24 |
| ***NfL*** | **1.20 (1.01;1.44)** | **0.04** | **1.21 (1.01;1.50)** | **0.04** | **1.21 (1.02;1.45)** | **0.03** | **1.17 (1.02;1.40)** | **0.03** | **1.18 (1.03;1.36)** | **0.02** | **1.18 (1.03;1.38)** | **0.02** |
| ***GFAP*** | 1.08 (0.94;1.25) | 0.27 | 1.09 (0.96;1.25) | 0.17 | 1.09 (0.96;1.25) | 0.19 | 1.02 (0.72;1.44) | 0.92 | 1.21 (0.80;1.62) | 0.54 | 1.19 (0.82;1.71) | 0.36 |

*Notes*: Model 1 is adjusted for age and education; Model 2 is additionally adjusted for smoking and alcohol habits, as well as comorbidities including diabetes, heart, cerebrovascular diseases, and chronic kidney disease, and physical activity; Model 3 is additionally adjusted for dietary variables (energy intake, protein intake, and adherence to Mediterranean diet).

**Supplementary Table 16. Cox regression sensitivity analyses examining the independent association between biomarkers and sarcopenia incidence, in people with dietary information, by age (≥78 and <78 years).**

|  | **Age <78 years (n=1304)** | | | | | | **Age ≥78 years (n=604)** | | | | | |
| --- | --- | --- | --- | --- | --- | --- | --- | --- | --- | --- | --- | --- |
|  | **Model 1** | | **Model 2** | | **Model 3** | | **Model 1** | | **Model 2** | | **Model 3** | |
|  | **OR**  **(95% CI)** | **p-value** | **OR**  **(95% CI)** | **p-value** | **OR**  **(95% CI)** | **p-value** | **OR**  **(95% CI)** | **p-value** | **OR**  **(95% CI)** | **p-value** | **OR**  **(95% CI)** | **p-value** |
| ***Aβ42/40*** | 1.02 (0.79;1.31) | 0.90 | 1.03 (0.80;1.32) | 0.85 | 1.02 (0.79;1.30) | 0.87 | **0.74 (0.57;0.96)** | **0.02** | **0.76 (0.59;0.98)** | **0.03** | **0.76 (0.59;0.97)** | **0.03** |
| ***t-tau*** | 0.94 (0.67;1.32) | 0.73 | 0.94 (0.70;1.32) | 0.73 | 0.95 (0.69;1.32) | 0.76 | 0.94 (0.76;1.17) | 0.60 | 0.93 (0.75;1.16) | 0.55 | 0.96 (0.77;1.19) | 0.68 |
| ***p-tau181*** | 1.35 (0.97;1.87) | 0.08 | 1.34 (0.95;1.91) | 0.10 | 1.34 (0.94;1.91) | 0.10 | **1.33 (1.10;1.61)** | **0.004** | **1.30 (1.07;1.58)** | **0.008** | **1.29 (1.07;1.60)** | **0.008** |
| ***NfL*** | 1.16 (0.99;1.37) | 0.07 | 1.17 (0.99;1.39) | 0.07 | 1.16 (0.97;1.37) | 0.10 | **1.16 (1.06;1.28)** | **0.002** | **1.16 (1.03;1.29)** | **0.01** | **1.18 (1.05;1.32)** | **0.005** |
| ***GFAP*** | 1.04 (0.87;1.24) | 0.67 | 1.05 (0.87;0.12) | 0.63 | 1.04 (0.87;1.24) | 0.68 | 1.10 (0.96;1.26) | 0.17 | 1.09 (0.97;1.24) | 0.14 | 1.09 (0.97;1.24) | 0.14 |

*Notes*: Model 1 is adjusted for sex and education; Model 2 is additionally adjusted for smoking and alcohol habits, as well as comorbidities including diabetes, heart, cerebrovascular diseases, and chronic kidney disease, and physical activity; Model 3 is additionally adjusted for dietary variables (energy intake, protein intake, and adherence to Mediterranean diet).
